# Promotion mechanism of high-involvement human resource management practices to employees’ bootlegging: A moderated mediation model

**DOI:** 10.3389/fpsyg.2022.1051420

**Published:** 2023-01-13

**Authors:** Jianfeng Jia, Zhi Liu, Weipeng Liu, Jieli Hu

**Affiliations:** ^1^School of Business Administration, Northeastern University, Shenyang, Liaoning, China; ^2^Renmin Business School, Renmin University of China, Beijing, China; ^3^Human Resources Department, Bank of Jiangsu Co., Ltd. Shenzhen Branch, Shenzhen, Guangdong, China

**Keywords:** high-involvement human resource management practices, bootlegging, psychological ownership, Chinese traditionality, stimuli-organism-response model

## Abstract

**Introduction:**

Bootlegging is a frontier topic in micro-innovation literature. Existing research on the external environment-antecedents of employees’ bootlegging focuses mainly on organizational innovation management practices and leadership. The relationship between human resource management and employees’ bootlegging is still unclear. Thus, we follow the stimuli-organism-response model and use psychological ownership theory to examine a moderated mediation model with psychological ownership as a mediator and Chinese traditionality as a moderator to interpret how and when high-involvement human resource management practices influence employees’ bootlegging.

**Methods:**

We administered three-wave time-lagged surveys to 251 employees and used SEM analysis to test the hypotheses.

**Results:**

The results show that high-involvement human resource management practices is positively related to employees’ psychological ownership. Whereas psychological ownership, in turn, positively related to bootlegging. Meanwhile, employees’ psychological ownership plays a significant mediating role between high-involvement human resource management practices and employees’ bootlegging. The results further showed that employees’ Chinese traditionality weakens the influence of psychological ownership on bootlegging and the mediating effect of employees’ psychological ownership between high-involvement human resource management practices and employees’ bootlegging.

**Discussion:**

This study makes several contributions to the bootlegging antecedent mechanism research. Specifically, it expands the understanding of the antecedents of bootlegging from a new perspective of human resource management, enriches the bootlegging-promotive cognition path from the perspective of psychological ownership, and enriches the proximal boundary in bootlegging antecedent mechanism from the perspective of individual personality. This study also inspires enterprises in innovation and talent management.

## 1. Introduction

Does innovation necessarily come from top-down planning? This view has been the mainstream for a long time (Globocnik et al., 2022). However, with market uncertainty and competition intensifying, scholars and managers have gradually realized that it is not always enough to create innovations to cope with environmental changes based only on management’s plans (Criscuolo et al., 2014). Therefore, the concept of “planned emergence” was proposed, reminding organizations to emphasize both top-down innovation design and bottom-up flexible micro-level innovation actions consistent with the organization’s goals (Prashantham and Eranova, 2020). In recent years, bootlegging as a bottom-up innovation action has become a hot research topic (Criscuolo et al., 2014; Jia et al., 2021; Li et al., 2021). It refers to an employee’s innovative behavior without managers’ knowledge and permission, which aims to benefit the organization (Augsdorfer, 2005; Criscuolo et al., 2014). Bootlegging is common in commercial organizations. For example, Wang Xiaochuan, the former CEO of SOHU, concealed the leadership and organized the R&D of Sogou browser, creating a valuation of tens of billions of dollars. Google tacitly gives consent to the investment of 20% employees’ working time to explore unauthorized projects, which has given birth to many phenomenal products such as AdSense and Gmail. As found in reality and emphasized in the concept of “planned emergence,” several studies have indicated the positive contribution of bootlegging to organizational innovation, such as improving innovation performance (Criscuolo et al., 2014), increasing the newness of the organization’s innovation portfolio (Globocnik et al., 2022), and so on. In this context, we should clarify why and when employees will conduct bootlegging, thereby providing more effective innovation management strategies.

The stimuli-organism-response (SOR) model indicates that the external environment shapes the individual’s cognition and leads to behavior (Vieira, 2013). Consistently, previous studies have shown that various external environmental factors, such as organizational innovation management practices, including emergent innovation initiatives, and leadership, like paradoxical leadership, have significant effects on activating employees’ bootlegging (Jia et al., 2021; Globocnik et al., 2022). However, human resource management practices (HRMP) are regarded as one of the core predictors of a series of employee behaviors (Chen et al., 2018; Hewagama et al., 2019), including innovative behavior (Lei et al., 2021), have been neglected in prior literature. Therefore, based on the SOR framework, we hope to discuss the relationship between HRMP and bootlegging to make bootlegging’s antecedents more comprehensive.

What sort of HRMP can affect employees’ bootlegging? From earlier studies, enhancing employees’ motivation and giving them autonomous innovation space are two important factors that promote bootlegging (Globocnik and Salomo, 2015; Sakhdari and Bidakhavidi, 2016; Jia et al., 2021). On these grounds, we expect that high-involvement human resource management practices (HI-HRMP) can be related to employees’ bootlegging. Specifically, HI-HRMP describes several human resource management practices that value employee participation and commitment, including the five aspects of recognition, empowerment, competence development, fair rewards, and information sharing (Ordiz-Fuertes and Fernández-Sánchez, 2003; Paré and Tremblay, 2007; Yang, 2012). In reality, many companies that highly value innovation have adopted one or more HI-HRMP methods to manage employees. For example, Xerox established a perfect electronic system to convey information to employees timely and designed a fair compensation incentive scheme to make employees have the main decision-making rights. The above typical practices strongly stimulated the innovation boom which helped Xerox gain an edge in the fierce international market competition.

Furthermore, some contents in HI-HRMP, such as helping employees improve their abilities and providing them with fair remuneration, can strengthen their motivation to benefit the organization through proactive behavior (e.g., bootlegging; Yang, 2012; Boxall et al., 2015). Meanwhile, the empowering practices of HI-HRMP can also give employees certain discretion (Paré and Tremblay, 2007) so that they can modify rules to bootleg secretly. Based on the above suggestion, this study explores the relationship between HI-HRMP and employees’ bootlegging.

In line with the SOR model, HI-HRMP alters employees’ bootlegging through a certain psychological cognition. However, the SOR model does not give us a universal cognition variable to explain how external stimuli change employee behavior. Thus, to explain the relationship between HI-HRMP and employees’ bootlegging, we further introduce the psychological ownership theory as the logical basis. Psychological ownership reflects employees’ subjective perception of the degree that they are the organization’s owner (Pierce et al., 2001, 2003; Avey et al., 2009). In past studies, psychological ownership was often incorporated into the SOR framework to bridge the stimuli of the external environment and employees’ behavioral responses. For instance, based on SOR, Lee and Yoo (2021) revealed how an organization’s internal market orientation promotes employees’ innovative behavior through psychological ownership.

Similarly, we propose that psychological ownership mediates between HI-HRMP and employees’ bootlegging. On the one hand, the psychological ownership theory suggests that individuals with strong psychological ownership will safeguard the profits of their “property” and hold a high sense of behavioral self-control (Pierce et al., 2001, 2003; Dawkins et al., 2017), which means employees with strong psychological ownership are more willing to bootleg for organizational gains and feel they have the autonomy to conduct bootlegging privately.

On the other hand, the psychological ownership theory also points out three psychological ownership-enhancing ways, that is, to let employees feel that their work activities in the organization are self-controlled, that they know key information about the organization, and that they devote time, effort, and attention to organizational goals (Pierce et al., 2001, 2003; Dawkins et al., 2017). Correspondingly, HI-HRMP meets such conditions. For example, HI-HRMP includes a typical practice of sharing information with employees (Paré and Tremblay, 2007). It is one of the direct inducements of psychological ownership mentioned above. Another study shows that HI-HRMP can enhance employees’ sense of control at work (Rana, 2015), which is positively related to employees’ psychological ownership. Moreover, HI-HRMP has been proven to make employees more engaged to work (Maden, 2015), while such engagement will also help strengthen psychological ownership. Therefore, we use psychological ownership as a mediator to make the impact process of HI-HRMP on employees’ bootlegging visible.

Prior SOR-based studies have also shown that the effect of cognition on behavior is often contingent on an individual’s characteristics (Wang et al., 2018; Joshua et al., 2022). This means that even based on the same psychological cognition, people with different personalities have different behavioral reactions. For example, Zhou and Long (2012) suggested that psychological ownership could trigger more employees’ voice behavior, but this relationship was weak among Chinese traditionalists. Xiong Chen and Aryee (2007) found that under the same level of organization-based self-esteem and perceived insider status, the more traditional an employee is, the weaker his innovation ability is. Following this thinking, we propose a personal characteristics moderator of Chinese traditionality to capture the borderline relationship between psychological ownership and employees’ bootlegging. Chinese traditionality refers to an individual value formed under the influence of Chinese culture (Farh et al., 1997, 2007). Because Chinese workers are widely distributed in various international business organizations, researchers have paid great attention to this concept (Li et al., 2017). Relevant studies suggested that employees with strong Chinese traditionality are more conformist and tend to abide by established policies and instructions from their superiors; they are less likely to take innovative or initiative behaviors under the same cognition conditions (e.g., affective commitment, psychological need satisfaction, and psychological ownership; Zhou and Long, 2012; Wang et al., 2020; Chen et al., 2021). However, bootlegging is such a creative activity that requires employees’ initiative (Jia et al., 2021). Hence, we infer that although employees with high psychological ownership are willing to carry out bootlegging, those with strong Chinese traditionality will conduct it less. We will investigate the moderating role of Chinese traditionality between psychological ownership and employees’ bootlegging and the related moderated mediation effect.

This study contributes to the literature in the following ways. First, based on the SOR model, we examined the relationships by which HI-HRMP affects bootlegging *via* psychological ownership. This expands our understanding of the antecedents of bootlegging from a new perspective of human resource management. Second, in prior bootlegging research, the psychological cognitive, which is often used to explain why employees engage in bootlegging, focuses mainly on self-efficacy and motivation (Globocnik and Salomo, 2015; Ghasemzadeh et al., 2021). Thus, by demonstrating our model, we enrich the bootlegging-promotive cognition path from the perspective of psychological ownership. Finally, previous bootlegging research has mainly responded to the question of why employees would conduct bootlegging and we still know little as to the circumstances why employees would do more or less. That is, the relationship boundary is not clear. In this regard, we propose that the employee whose bootlegging is more likely to be promoted by psychological ownership strengthened by HI-HRMP if he has a weak Chinese traditionality. This view enriches the proximal boundary in bootlegging antecedent mechanism from the perspective of individual personality.

This study’s structure is as followed. In section 2, based on the stimuli-organism-response model and psychological ownership theory, we develop a moderated mediation model to explain the relationship between HI-HRMP and employees’ bootlegging which is consisted of five hypotheses. In sections 3 and 4, we introduce the research methodology and analysis results. In section 5, we summarize the research conclusions and discuss the theoretical and practical contributions as well as limitations and future research directions.

## 2. Theory and hypotheses

### 2.1. HI-HRMP and psychological ownership

Psychological ownership is the core concept of the psychological ownership theory. It describes the mentality in which an individual feels that the object belongs to them. In organization situation-based research, psychological ownership is usually used to indicate the degree to which employees think they are the organization’s owners (Pierce et al., 2001, 2003). Psychological ownership includes four sub-constructs (Avey et al., 2009): self-efficacy, that is, employees are confident that they can successfully perform work tasks and achieve work goals; belongingness, that is, employees’ sense of belonging to the organization; self-identity, which refers to the degree of which employees perceive that the organization and work is the extension and expression of themselves; and accountability, that is, the extent to which employees think they should be responsible for the organization and work, and actively share the pressure for the organization. Drawing on the SOR model, the external environment is key to shaping employees’ cognition (e.g., psychological ownership; Vieira, 2013; Lee and Yoo, 2021). In this study, we used HI-HRMP to capture such an external environment. HI-HRMP is a collection of employee participation-oriented human resource management practices, including four factors, namely empowering employees, supporting employees to develop competence, providing fair rewards, and sharing information with employees (Paré and Tremblay, 2007; Yang, 2012). Based on the psychological ownership theory, we propose that HI-HRMP positively relates to employees’ psychological ownership.

First, the psychological ownership theory advised that if environmental conditions endow individuals with the opportunity to exercise and experience control, their psychological ownership will be triggered (Furby, 1978; Pierce et al., 2001; Dawkins et al., 2017). To explain this view in the organizational context, employees’ control sense in the organization can boost their perception of being quite capable and influential (Avey et al., 2009), elevating their confidence in completing work tasks and organizational goals. Also, employees who are in control of their work may realize that they can fully express their views and desires at work which strongly strengthens their self-identity (Kim and Beehr, 2017). That is, employees’ psychological ownership dimensions of self-efficacy and self-identity are effectively loaded. HI-HRMP could be a shaper of such control sense because it emphasizes empowering employees to strengthen their participation in the organization’s decision-making process (Paré and Tremblay, 2007). In turn, employees feel that they can control their work content and goals to a certain extent. And it is not difficult to express themselves in the organization and to influence organizational affairs according to their opinions (Coun et al., 2022). Meanwhile, HI-HRMP vigorously supports employees to develop their abilities, not only by offering courses to help employees learn vocational work knowledge but also providing professional expansion activities (e.g., counseling and training) to improve their skills (Yang, 2012). Over time, employees can cope with the tasks, problems, and challenges more smoothly and perceive stronger work and organization-related control and efficacy.

Second, in the light of the psychological ownership theory (Pierce et al., 2001, 2003), deepening employees’ understanding of the organization, especially allowing employees to grasp organizational information related to themselves (such as performance, plans, and goals) will help them establish psychological ownership. This is because, at the psychological level, the more information individuals know about an object, the more likely they are to have an “owner” identity cognition and develop stronger belongingness and responsibility to the object which is the foundation of an organization’s self-identity (Furby, 1978; Zhang et al., 2021). Meanwhile, in-depth understanding, to a certain extent, also can be deemed as the result of an individual’s exploration and control of a target, and thus, mastery of information can also improve an individual’s self-efficacy in relevant matters (Furby, 1978). Correspondingly, HI-HRMP includes a series of practices to share information with employees, such as regularly informing employees of the organization’s key activities (e.g., large-scale investment, mergers and acquisitions), management rules, financial position, and department performance (Yang, 2012). Thus, HI-HRMP can increase employees’ psychological ownership by consolidating belongingness and accountability to the organization, self-identity, and self-efficacy of work.

Finally, the psychological ownership theory points out that individuals have stronger psychological ownership of the objects they invest more time, energy and resources in because the investment not only makes them feel that, to a certain extent, that the object is produced by themselves, they own it and be responsible for it, but also gives individuals a carrier to place themselves, thereby meeting their needs of self-cognition (Pierce et al., 2001). On these grounds, we suggest that HI-HRMP can intensify the organic components of employees’ psychological ownership: a sense of belongingness, accountability, and self-identity. Concretely, by emphasizing the empowerment of employees, HI-HRMP allows employees to express their self-will in organizational decision-making and to deeply participate or even lead the work implementation (Boxall and Huo, 2022). It causes employees to realize that they have a goal unity with the organization and are key internal members of the organization (Gahlawat and Kundu, 2020). This realization in turn, will strengthen employees’ sense of belonging and self-identity. HI-HRMP also values the cultivation and development of employees and designs a fair salary scheme for employees (Yang, 2012). In return, employees will be more involved in their work and have a stronger sense of responsibility for the organizational goals (Hartmann and Slapničar, 2012; Meng and Wu, 2015). Thus, we propose the following hypothesis:

*Hypothesis 1*: HI-HRMP is positively related to employees’ psychological ownership.

### 2.2. Mediating role of psychological ownership

In line with the SOR model, after the stimulation of the external situation shapes the individual’s psychological cognition, it will further change the employee’s behavior (Vieira, 2013). Based on the SOR model and psychological ownership theory, we consider that when employees’ psychological ownership is enhanced from the interaction with HI-HRMP, they have a larger probability of carrying out bootlegging. Bootlegging is generally considered to be a certain constructive deviant or innovative behavior, and describes the innovative behavior that employees secretly conduct to benefit the organization without formal authorization (Criscuolo et al., 2014). Given that bootlegging naturally has a higher risk attribute like most creative acts and that it is usually carried out without the permission and support of the superior or the organization (Jia et al., 2021), enhancing employees’ motivation to benefit the organization and dispelling their worries about behavioral risk are regarded as two significant paths that affect employees’ bootlegging in former literature (Globocnik and Salomo, 2015; Sakhdari and Bidakhavidi, 2016; Jia et al., 2021). In this study, we hold that psychological ownership combines the logic of these two paths, thus acting on bootlegging. Specifically, on the one hand, in the light of psychological ownership theory, employees with strong psychological ownership regard themselves as the masters of the organization, which means they have a strong sense of belonging and responsibility for the organization (Avey et al., 2009). Thus, they are willing to take the initiative to engage in all activities that can safeguard and even create the organization’s interests, including conducting bootlegging. Meanwhile, employees’ self-identity based on the organization will also appear accompanied by psychological ownership (Xia et al., 2019), which makes employees believe they are consistent with the organization in terms of goals and that their self-cognition is integrated into the organization’s values, thereby promoting the organization’s progress to make themselves better. Thus, for those innovative ideas with latent capacity, even without organizational requirements, arrangements, and authorizations, the employees also have sufficient motivation to take risks to practice them in the form of private bootlegging.

On the other hand, psychological ownership theory suggests that self-efficacy is the core dimension of psychological ownership, which reflects employees’ sense of control and confidence based on organization and work (Avey et al., 2009). First, employees with high psychological ownership perceive a certain degree of discretion with which they can decide a part of their work autonomously and exert influence on organizational affairs (Zhang et al., 2021). In this case, employees tend to deem that even without the organization’s authorization, their private bootlegging is tacitly approved and will not be punished in the future (Jia et al., 2021). Second, the self-efficacy among psychological ownership also gives employees confidence even without organizational resource support and program guidance. Hence, psychological ownership dispels employees’ concerns about bootlegging, encouraging them to engage in more bootlegging behavior. We propose the following hypothesis:

*Hypothesis 2*: Employees’ psychological ownership is positively related to bootlegging.

We synthesize the hypotheses consistent with the framework of the SOR Model. First, HI-HRMP can enhance employees’ psychological ownership through the three classical logic suggested by the psychological ownership theory. Then, the raised psychological ownership further promotes employees’ bootlegging by intensifying employees’ behavioral motivation and eliminating their worry about bootlegging’s difficulties and risks. Hence, we propose the following hypothesis:

*Hypothesis 3*: Employees’ psychological ownership mediates the relationship between high-involvement human resource management and employees’ bootlegging.

### 2.3. Moderating role of Chinese traditionality

Although no direct discussion on the relationship boundary between individual psychological cognition and behavior in the SOR model is available, several SOR-based studies indicate that the influence degree of individual psychological cognition on their behavior could often be moderated by individual characteristics (Wang et al., 2018; Joshua et al., 2022). Similarly, this study proposes that psychological ownership is likelier to stimulate bootlegging for employees with weaker Chinese traditionality. Chinese traditionality is one of the most typical personalities of Chinese people, derived from Chinese traditional culture, such as the golden mean (Cheung et al., 1996; Farh et al., 1997, 2007). Chinese traditionality includes obeying authority (i.e., unconditional compliance with authority and rules), remaining in one’s proper sphere (i.e., rejecting to work crossing levels or breaking the rules), fatalism (i.e., conservatively facing the risks of initiative and change) and so on (Farh et al., 1997, 2007). These factors can weaken the influence of psychological ownership on bootlegging.

On the one hand, employees with strong Chinese traditionality will follow the golden mean to launch their work and behavior conservatively (Farh et al., 2007). They often rarely pursue transformation, exploration, and innovation, nor are they unwilling to do things beyond their duties but tend to achieve the work goals arranged by the organization following the prescribed order (Hu et al., 2020; Chen et al., 2021). However, as an out-of-role behavior, bootlegging is rarely approved by the organization and is full of risks (Mainemelis, 2010; Criscuolo et al., 2014; Jia et al., 2021). Hence, even if psychological ownership strengthens the motivation to make profits for the organization, employees tend to implement specific actions within their responsibilities rather than the bootleg.

On the other hand, the influence of Chinese traditionality means that employees consistently abide by vested rules and systems, obey the orders of managers, and have weak flexibility and initiative in decision-making and behavior (Farh et al., 1997; Yang, 2012). Given that bootlegging is not the product of the instructions of leaders and even generally violates organizational rules (Augsdorfer, 2005; Criscuolo et al., 2014), even if the employees with stronger psychological ownership have an improved sense of work control and self-efficiency, they are still unlikely to go beyond the rules of their superiors and organizations to conduct bootlegging in compliance with their own decisions. Thus, strong Chinese traditionality undermines the connection between employees’ psychological ownership and bootlegging.

By contrast, employees with weak Chinese traditionality are not stick-in-the-muds (Xu et al., 2021). They have no objection to carry out creative activities such as bootlegging to change the status quo which are bound to exceed their scope of work (Hu et al., 2020). As employees’ motivation to benefit the organization increases under the influence of psychological ownership, they are more likely to engage in bootlegging actively which is supererogatory for their job duties but helps the organization to find latent opportunities.

At the same time, employees with weak traditionality in China are only willing to obey the organization’s institution and instructions from superiors under certain circumstances and they prefer to work flexibly according to their own ideas (Chen et al., 2021; Wang, 2022). Hence, when high psychological ownership brings employees a sense of control and self-efficacy in their work, those who have a weak Chinese traditionality are more likely to implement their bootlegging ideas privately rather than temporarily giving up innovation and awaiting orders from the organization or leader. To sum up, we propose the following hypothesis:

*Hypothesis 4*: Employees’ Chinese traditionality negatively moderates the relationship between employees’ psychological ownership and employees’ bootlegging.

Because Chinese traditionality weakens the effects of employees’ psychological ownership and employees’ bootlegging, and employees’ psychological ownership mediates the effects of HI-HRMP on bootlegging, we propose the following moderated mediation hypothesis:

*Hypothesis 5*: Employees’ Chinese traditionality negatively moderates employees’ psychological ownership’s mediating effect between high-involvement human resource management and employees’ bootlegging.

**Figure 1:**
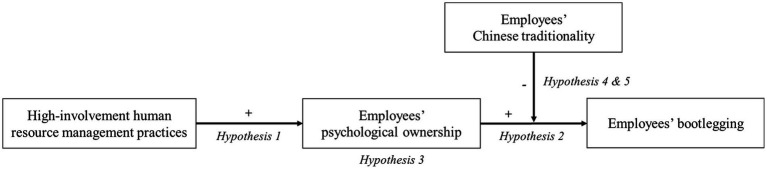
Theoretical model. “+” and “−,” respectively, represent the positive and negative relationship.

Our conceptual model hypothesized is presented in Figure 1.

## 3. Research methodology

### 3.1. Participants and procedure

Survey data were collected from 361 full-time employees in a manufacturing company’s headquarters in southern China and two branches in southern and northern China, respectively. Participants are distributed in the R&D, design, strategic, marketing, finance, manufacturing and other departments. We used a three-wave time-lagged survey design because the temporal separation between the independent, mediation, and dependent variables allowed previously recalled information to leave short-term memory, thereby reducing common method bias (Podsakoff et al., 2003). The participants who completed the survey received 50–200 RMB (approximately US$7–30) as a reward.

For preciseness and confidentiality, all surveys used paper questionnaires. In line with the general research ethics, all participants gave informed consent to participate in the study before the survey. Meanwhile, to ensure that the data of different waves can be matched accurately, we invited an executive of the HR department as a contact person. We asked them to provide unique but not private information about participants that allowed us to develop identification codes to match the data.

At Time 1, employees rated their perceived HI-HRMP and control variables (i.e., age, gender, educational background, and tenure); 1 month later (i.e., Time 2), employees who effectively completed Time 1’s questionnaire reported their psychological ownership and Chinese traditionality. At Time 3, 1 month after Time 2, employees who effectively completed Time 2’s questionnaire were asked to assess their own bootlegging. A total of 327 subordinates returned questionnaires at Time 1 (response rate of 90.58%), 291 at Time 2 (response rate of 89.04%), and 267 employees at Time 3 (response rate of 91.75%).

Participants were eligible if they had none of the following cases: (a) missing first-, second-, third- time data or a majority of blanks; (b) giving most or even all items the same scores; and (c) answering regularly but illogically (e.g., in a ladder shape). Finally, the questionnaires completed by 251 employees were chosen. Among the final 251 employees, 51.79% were male; 7.17% had an associate degree, 38.28% had a bachelor’s degree, 45.37% had a master’s degree, and 9.18% had a doctor’s degree; the average age was 34.92 (SD = 5.22).

### 3.2. Measures

Unless otherwise noted, variables were measured by a seven-point Likert scale (from 1 = strongly disagree to 7 = strongly agree). Participants largely identified as Chinese, and thus, we translated English-established measure items into Chinese using accepted Brislin’s (1980) translation-back-translation procedure. Table 1 contained a complete list of items.

**Table 1:** Results of the reliability analysis (*N* = 251).

Variables	Items	Loading	Cronbach’s alpha	AVE	CR
HI-HRMP (Yang, 2012)	1. When I do good quality work, my colleagues regularly show me their appreciation	0.596	0.928	0.553	0.930
	2. In my work unit, supervisors tangibly recognize my efforts in different ways	0.745			
	3. In my work unit, supervisors regularly congratulate me in recognition of my efforts	0.794			
	4. We are given great latitude for the organization of our work	0.739			
	5. In my work unit, we have considerable freedom regarding the way we carry out our work	0.801			
	6. We can develop our skills in order to increase our chances of being promoted	0.768			
	7. We can rotate jobs to develop our skills	0.730			
	8. Several professional development activities (e.g., coaching, training) are offered to us to improve our skills and knowledge	0.702			
	9. I estimate my salary as being fair internally	0.743			
	10. My salary is fair in comparison with what is offered for a similar job elsewhere	0.752			
	11. In my work unit, we consider that our compensation level adequately reflects our level of responsibility in the organization	0.740			
	12. We are regularly informed of financial results	0.791			
	13. We are regularly informed of our work unit’s performance	0.697			
Psychological ownership (Avey et al., 2009)	1. I am confident in my ability to contribute to my organization’s success	0.627	0.803	0.506	0.913
	2. I am confident I can make a positive difference in this organization	0.660			
	3. In the organization, I am confident in setting high performance goals	0.645			
	4. I would challenge anyone in my organization if I thought something was done wrong	0.836			
	5. I would not hesitate to tell my organization if I saw something that was done wrong	0.632			
	6. I will query the development direction of the organization to ensure if it is correct or not	0.596			
	7. I feel I belong in this organization	0.756			
	8. For me, organization makes me feel just at home	0.768			
	9. I am totally comfortable being in this organization	0.738			
	10. I feel this organization’s success is my success	0.677			
	11. I feel being a member in this organization helps define who I am	0.724			
	12. When the organization is criticized, I feel it is necessary to defend it	0.823			
Chinese traditionality (Farh et al., 1997)	1. The best way to avoid mistakes is to follow the instructions from senior persons	0.754	0.895	0.718	0.927
	2. Even if the superior’s request is unreasonable, we still should follow it	0.891			
	3. Even if your work is not satisfactory, you still work hard and comply with work arrangement	0.904			
	4.The leader is like the patriarch	0.849			
	5. When people are in dispute, they should ask the person with the highest rank to decide who is right	0.830			
Bootlegging (Criscuolo et al., 2014)	1. I have the flexibility to work my way around my official work plan, digging into new potentially valuable business opportunities	0.689	0.814	0.539	0.853
	2. My work plan does not allow me the time to work on anything other than the projects I have been assigned to	0.782			
	3. I enjoy tinkering around with ideas that are outside the main projects I work on	0.801			
	4. I am running several pet projects that allow me to learn about new areas	0.689			
	5. I proactively take time to work on unofficial projects to seed future official projects	0.699			

(a) AVE is the abbreviation of “Average variance extracted”; (b) CR is the abbreviation of “Composite reliability.”

**HI-HRMP**. A 13-item scale developed by Yang (2012) in Table 1 was adopted. Cronbach’s alpha was *α* = 0.928.

**Psychological ownership**. A 12-item scale developed by Avey et al. (2009) was adopted. Cronbach’s alpha was *α* = 0.803.

**Chinese traditionality**. A five-item scale developed by Farh et al. (1997) was adopted, and we made a fine adjustment in combination with the organizational situation and Chinese language habits. Cronbach’s alpha was *α* = 0.895.

**Bootlegging**. A five-item scale developed by Criscuolo et al. (2014) was adopted. Cronbach’s alpha was *α* = 0.814.

**Control variables**. We followed previous literature on bootlegging (Jia et al., 2021) and controlled for the possible confounding effects of employees’ age (in years), gender (0 = male; 1 = female), educational background (0 = associate’s degree, 1 = bachelor’s degree, 2 = master’s degree, 3 = doctor’s degree) and tenure (in Months).

## 4. Results

### 4.1. Test of reliability

Dual statistics are used to assess reliability. At the construct level, internal consistency (captured by Cronbach’s alpha) is presented in Table 1, where all values are greater than 0.8. Second, at the model level, average variance extracted (AVE) and composite reliability (CR) is also presented in Table 1, where all values of average variance extracted are greater than 0.5 and all values of composite reliability are greater than 0.7. The results show that the measures are reliable.

### 4.2. Validity of the constructs

Given the large number of measurement items (35 items in total), we use the item parceling approach to reduce the ratio of variables to sample size (Landis et al., 2000; Little et al., 2002). Parceling is suggested for non-normality issues and is considered applicable when a study focuses on the relations among variables rather than the relations among items representing a latent variable (Little et al., 2002; Williams and O’Boyle, 2008). Therefore, we applied this approach to all latent variables. Specifically, we used the item-to-construct balance method to create parcels. This method suggested assigning the item with the highest factor loading to the first parcel, the second highest to the second parcel, and so forth (Landis et al., 2000; Williams and O’Boyle, 2008), through which we created four parcels for HI-HRMP and psychological ownership, and two parcels for bootlegging and Chinese traditionality, respectively.

We used Mplus 7.0 for confirmatory factor analysis (CFA) to test the discriminant validity of the main variables. Table 2 shows that all the fit indices of hypothesized four-factor model were accepted (*χ*^2^ = 69.695; df = 48; CFI = 0.988; TLI = 0.984; RMSEA = 0.042; SRMR = 0.028) and better than other alternative models. The result indicated a good discriminant validity among HI-HRMP, psychological ownership, bootlegging, and Chinese traditionality.

**Table 2:** Results of confirmatory factor analysis (*N* = 251).

Models	*χ* ^2^	df	Δ*χ*^2^ (df = 1)	CFI	TLI	RMSEA	SRMR
Four-factor model + CMV	39.597	37	2.736	0.999	0.997	0.017	0.019
Four-factor model	69.695	48	–	0.988	0.984	0.042	0.028
Three-factor model	276.604	51	68.970***	0.877	0.841	0.133	0.091
Two-factor model	730.729	53	227.063***	0.631	0.541	0.226	0.176
One-factor model	1035.752	54	305.023***	0.466	0.347	0.269	0.196

(a) ***p < 0.001; (b) CMV is the abbreviation of “common method variance factor which predicted by all observed variables”; (c) Three-factor model: combing HI-HRMP and bootlegging as one factor; Two-factor model: combing HI-HRMP, psychological ownership and bootlegging as the other factor.

### 4.3. Common method bias test

Although we have adopted a time-lagged design, due to the invariance of the respondents, common method bias may still exist, and thus, we test common method bias using the method of adding the common method variance (CMV) to the four-factor model, which is suggested by Podsakoff et al. (2003). Table 2 shows that the result showed the fitting indices have minor improvement (Δχ^2^/Δdf = 2.736, n. s.). That is, CMV does not significantly exist in our data.

### 4.4. Descriptive statistic

Correlation analysis for the link of variables in this study was conducted as the initial results. As shown in Table 3, HI-HRMP was positively related to employees’ psychological ownership (*r* = 0.188, *p* < 0.01) and bootlegging (*r* = 0.265, *p* < 0.01); employees’ psychological ownership was positively related to employees’ bootlegging (*r* = 0.257, *p* < 0.01). Means, standard deviations, and the Cronbach’s alpha of all variables are also presented in Table 3.

**Table 3:** Descriptive statistics and correlation (*N* = 251).

Variables	1	2	3	4	5	6	7	8
1. Age	–							
2. Gender	0.094	–						
3. Education background	−0.080	0.127*	–					
4. Tenure	−0.036	−0.061	−0.117	–				
5. High-involvement human resource management practices	−0.027	−0.006	0.044	−0.023	(0.928)			
6. Psychological ownership	−0.089	0.093	0.028	0.123	0.188**	(0.803)		
7. Bootlegging	−0.013	0.038	−0.053	−0.011	0.265**	0.257**	(0.895)	
8. Chinese traditionality	−0.457**	−0.087	−0.041	−0.060	−0.114	−0.029	−0.155*	(0.814)
Mean	0.518	34.920	1.566	60.111	5.094	5.201	3.578	5.360
SD	0.501	5.220	0.758	44.164	0.906	0.681	1.135	1.049

(1) Tests were two-tailed; (2) **p < 0.01 and *p < 0.05; (3) Cronbach’s alpha is provided in parentheses on the diagonal.

### 4.5. Hypotheses test

Table 4 depicts the overall model’s structural equation model (SEM) analysis results. The results were obtained after controlling for employees’ age (*β* = −0.038, SE = 0.161, ns), gender (*β* = 0.003, SE = 0.013, ns), educational background (*β* = −0.082, SE = 0.091, ns), and tenure (*β* = −0.001, SE = 0.002, ns). To estimate whether an improvement in the model fit of the overall model compared to the null model (i.e., partial mediation model), we utilized the Akaike Information Criterion (AIC) and the sample size adjusted Bayesian Information Criterion (BIC) suggested by Kline (2011), where lower values indicate a better model fit. The results show that the overall model fits the data better (ΔAIC = 13.156; ΔBIC = 12.802). Hence, we reported on findings from the overall model.

**Table 4:** Path coefficient of structural equation model (*N* = 251).

Hypothesized path	Estimated effect	Standard deviation	*T*-value	*p*-value	Test results
Hypothesis 1: HI-HRMP → employees’ psychological ownership	0.138**	0.048	2.853	0.004	Support
Hypothesis 2: employees’ psychological ownership → employees’ bootlegging	0.361**	0.126	2.872	0.004	Support
Hypothesis 3: HI-HRMP → employees’ psychological ownership → employees’ bootlegging	0.050*	0.024	2.063	0.039	Support
Hypothesis 4: employees’ psychological ownership * employees’ Chinese trait → employees’ bootlegging	−0.479***	0.129	−3.716	0.000	Support
Hypothesis 5: moderated mediation effect	−0.066**	0.029	−2.323	0.020	Support

***p < 0.001, **p < 0.01 and *p < 0.05.

The SEM results showed that HI-HRMP significantly positively affected employees’ psychological ownership (*β* = 0.138, SE = 0.048, *p* < 0.05), supporting Hypothesis 1. Employees’ psychological ownership had a significant positive effect on employees’ bootlegging (*β* = 0.361, SE = 0.126, *p* < 0.01), supporting Hypothesis 2. Employees’ psychological ownership had a significant mediation effect between HI-HRMP and employees’ bootlegging (*β* = 0.050, SE = 0.126, *p* < 0.01), Hypothesis 3 could be preliminarily supported. The interaction of employees’ psychological ownership and Chinese traditionality significantly negatively affected employees’ bootlegging (*β* = −0.479, SE = 0.129, *p* < 0.001), supporting Hypothesis 4. Finally, the product of the effect of HI-HRMP on employees’ psychological ownership and the effect of the interaction of employees’ psychological ownership and Chinese traditionality on employees’ bootlegging is significant (*β* = −0.066, SE = 0.029, *p* < 0.05), preliminarily supporting Hypothesis 5.

To further estimate the mediation effect in Hypothesis 3, we calculated the indirect effect using a bootstrapping procedure with 20,000 Monte Carlo replications, which can overcome the challenge of the non-normal distribution of samples in a certain. The results indicated that the indirect effect mediated by employees’ psychological ownership was 0.050 (SE = 0.024, 95% CIs [0.002, 0.090] excluded 0, *p* < 0.05), thereby providing stronger support for Hypothesis 3. Meanwhile, HI-HRMP’s direct effect on employees’ bootlegging is also significant (*β* = 0.318, SE = 0.083, *p* < 0.001), indicating that employees’ psychological ownership is a partial mediator.

**Figure 2:**
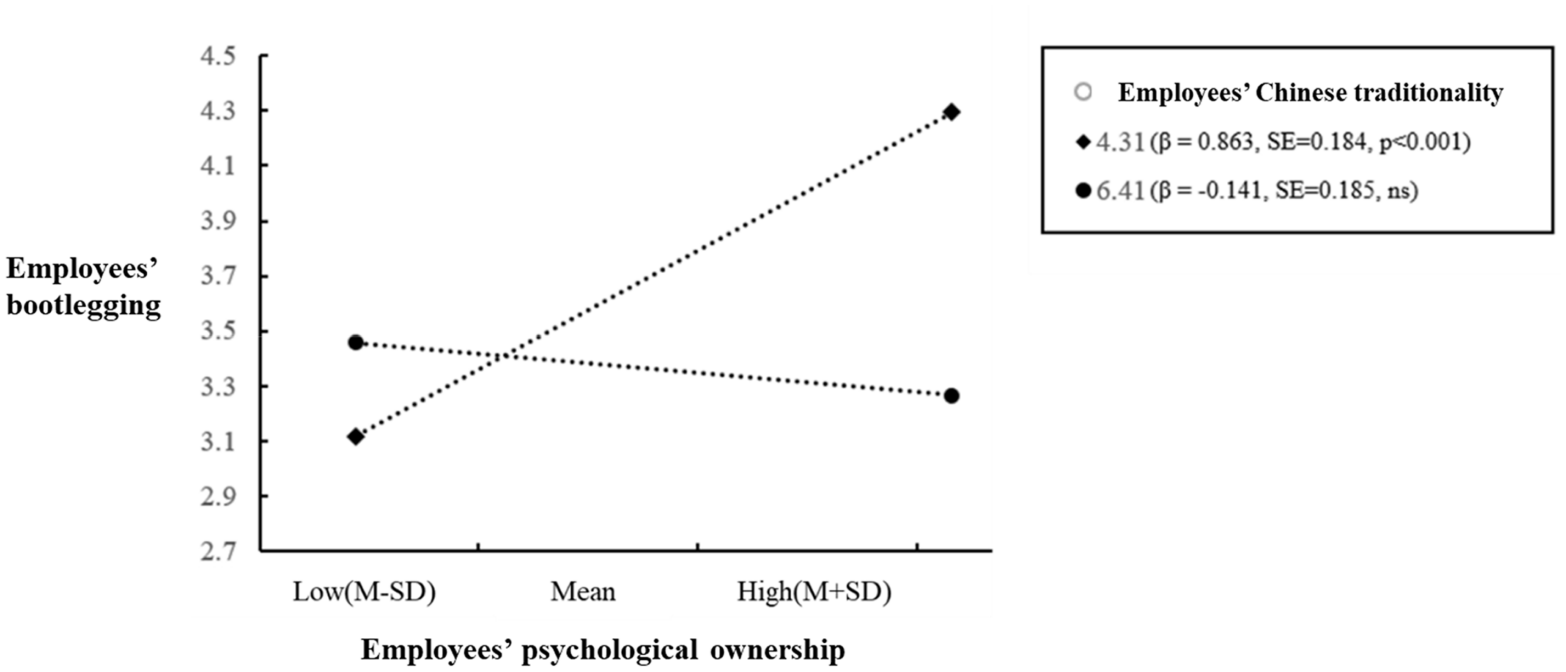
Moderating effect of Chinese traditionality on the relationship between psychological ownership and bootlegging.

To show the moderation effect proposed in Hypothesis 4 more intuitively, we plotted a simple slope analysis recommended by Aiken and West (1991), see Figure 2. As expected, employees’ psychological ownership was more positively correlated with employees’ bootlegging when employees’ Chinese traditionality was low (*β* = 0.863, SE = 0.184, *p* < 0.001) than when employees’ Chinese traditionality was high (*β* = −0.141, SE = 0.185, ns), with a significant difference in the relationship magnitude (Δ*β* = 1.005, SE = 0.270, *p* < 0.001).

Moreover, we followed Hayes (2015) to further examine the moderated-mediation effect in Hypothesis 5. Specifically, we tested the difference of the conditional indirect effect of employees’ psychological ownership under low and high levels of employees’ Chinese traditionality. On the one hand, the indirect, positive effect of HI-HRMP on employees’ bootlegging *via* psychological ownership was weaker when employees’ Chinese traditionality was high (*β* = −0.020, n. s.) than when employees’ Chinese traditionality was low (*β* = 0.119, SE = 0.048, *p* < 0.05), and with a significant difference in the relationship magnitude (difference = −0.139, SE = 0.060, *p* < 0.05). Thus, Hypothesis 5 was supported.

## 5. Discussion

### 5.1. Conclusion

This study focuses on revealing the promotion mechanism of HI-HRMP on employees’ bootlegging. Based on SOR model and psychological ownership theory, the findings indicate that HI-HRMP can strengthen employees’ psychological ownership, thereby activating employees’ bootlegging. Moreover, when it comes to employees with strong (weak) Chinese traditionality, the relationship between psychological ownership and bootlegging is weaker (stronger) and the impact of HI-HRMP on employees’ bootlegging through the mediator of employee psychological ownership is weaker (stronger). In all, we have provided new insights on how, why and when HI-HRMP affects employees’ bootlegging, we expect it can help organizations formulate innovation management policies better.

### 5.2. Theoretical implications

This study makes several contributions to innovation research, especially in the informal innovation literature. First, this study expands understanding of the antecedents of employees’ bootlegging from the perspective of HRM. Specifically, employee innovation is one of the hottest research topics in academia, but most studies focus on proper innovative behavior. Bootlegging, as an informal, innovative behavior, has been proven to have a positive effect on organizational innovation performance and ability as compared to that proper innovative behavior (Criscuolo et al., 2014; Globocnik et al., 2022), but our knowledge of its antecedents is still limited. The SOR model suggests that stimulating the external environment is the key inducement for individual behavior (Joshua et al., 2022). However, the previous studies focused only on organizational innovation management practices and leadership’s effect on employees’ bootlegging (Jia et al., 2021; Globocnik et al., 2022). HRM, as one of the most direct external interaction situations of employees at work and its connection with bootlegging was still unclear. Hence, by clarifying HI-HRMP’s improving psychological ownership-based influence on employees’ bootlegging, we are approximately the first to extend the booster of bootlegging to HRM factors.

Second, this study enriches the driving cognition mechanism of employee bootlegging from the perspective of psychological ownership. According to the SOR model, psychological cognitions are momentous proximal reasons for employees to show more certain behaviors (Vieira, 2013). However, as mentioned above, explorations of the antecedents of employee bootlegging are still limited and studies seldom discuss the triggering mechanisms of bootlegging. Against this background, the past known cognition factors related to promoting employees’ bootlegging are incomplete, mainly focusing unilaterally on self-efficacy (Globocnik and Salomo, 2015; Ghasemzadeh et al., 2021) and motivation. Hence, by using the SOR model and psychological ownership theory, we proposed the mediating role of psychological ownership between HI-HRMP and bootlegging, thereby adding novel theoretical insights to the interpretation mechanism of why employees would engage in bootlegging.

Finally, we construct a moderated mediation model, thereby answering the question of when does psychological ownership have stronger influence on employees’ bootlegging, and tentatively revealing what peculiarity makes employees more likely to be inspired to show bootlegging. In detail, as far as we know, existing literature hardly discusses the contingency factors of the emergence of bootlegging. In this study, we tested employees’ Chinese traditionality as a moderator to capture the boundary condition between employees’ psychological ownership and bootlegging. We find that the stronger employees’ Chinese traditionality, the weaker the influence of psychological ownership on employees’ bootlegging. This deepens our theoretical understanding of employees’ bootlegging and reminds future researchers to pay more attention to the role of individual traits in studies on the formation mechanism of bootlegging.

### 5.3. Practical implications

This research is enlightening for organizational innovation management and talent management. First, we found that HI-HRMP can help activate employees’ positive cognition of psychological ownership and innovative behavior of bootlegging, and thus, organizations should promote the application of HI-HRMP mode. Based on the definition of HI-HRMP and the logical demonstration of our hypothesis, we suggest that organizational human resource management should integrate content from multiple perspectives to strengthen employee involvement. In the HRM process, managers should reasonably express recognition and appreciation to employees, help employees strengthen their work ability, and share information with them in due time to ensure their right to know. Meanwhile, the organization should establish an appropriate employee authorization mechanism, develop employees’ ability and quality through a series of training projects, afford opportunities for employees to take self-growth, and design a fair reward scheme to motivate employees.

Second, we confirmed that strengthening psychological ownership is an effective way to trigger employees’ bootlegging, and hence, we advocate that organizations take measures to improve employees’ psychological ownership. We further suggest that the organization formulate appropriate incentives to make employees feel the benefits brought by work, strengthen employees’ sense of work control through some means (e.g., authorization, skill training), improve employees’ participation in work decisions to enhance their self-identity, and share information with employees to safeguard their right to know about work affairs, thereby making employees have a greater sense of belonging and responsibility to the organization.

Third, according to this study, driven by the same level of psychological ownership, more bootlegging occurs when employees have stronger Chinese traditionality. This conclusion can be applied to the development of an innovative team. While recruiting talents for innovative groups, the organization should emphasize employees’ values and select employees with low Chinese traditionality. In an organizational human resource development or talent training, more thought guidance must be launched to dilute employees’ fear of innovation and superstition about authority, which lurks under Chinese traditionality. For example, the organization should emphasize the importance of innovation and state that the risk of innovation is primarily borne by the organization. At the same time, the organization should cultivate employees’ awareness of equal rights between superiors and subordinates and encourage them to express their views bravely which can make them realize that they will not be criticized even if they take another view.

### 5.4. Limitations and future research perspectives

This study is not without limitations. First, based on the SOR framework, this study uses HI-HRMP to capture the external stimuli that affect employees’ bootlegging and interprets the linkage mechanism between them. However, although HI-HRMP is relevant in forecasting employees’ bootlegging, it is not necessarily the only choice. For example, high-performance HRM can also shape employees’ behaviors, including creative behaviors, by strengthening employees’ psychological cognitions, such as commitment and motivation (Fabi et al., 2015; Dorta-Afonso et al., 2021). Thus, in the future, more HRMP elements, such as high-performance HRMP, should also be examined as to whether they can join the antecedent network of bootlegging based on the SOR model.

Second, drawing on the SOR model and psychological ownership theory, we use psychological ownership as a bridge to establish the relationship between HI-HRMP and employees’ bootlegging. Still, we do not rule out the possibility of other suitable connectors between HI-HRMP and employees’ bootlegging. For instance, Hassan and Din’s (2019) research points out that HI-HRMP can enhance employees’ creativity by strengthening their intrinsic work motivation. Criscuolo et al. (2014) found that creativity is an important incentive for employees to show more bootlegging. Based on these research findings, we speculate that factors such as creativity may also mediate the effects of HI-HRMP on employees’ bootlegging, which can be verified in the future.

Third, this study tested the moderation effect of employees’ Chinese traditionality on the relationship between psychological ownership and employees’ bootlegging and tentatively answered what kind of employee’s bootlegging is more likely to be activated, but it is far from enough. Specifically, Chinese traditionality is only one of the individual characteristics that can modify the relationship strength of psychological ownership and employee bootlegging. Other variables also have similar logical potential. For example, compared with employees whose regulatory focus is biased toward prevention, employees with a promotion regulatory focus have a stronger innovation self-efficacy and less fear of risks (Crowe and Higgins, 1997), which means that they are more likely to implement bootlegging.

Fourth, this study is based on the premise that enterprises should encourage employees’ bootlegging. However, some recent scholars began to express concern about the dark side of employee bootlegging. For example, research suggests that employees’ bootlegging may break organizational rules, and bootlegging’s occupation of employees’ time may reduce their performance (Hooi and Tan, 2021). Therefore, coming studies may take the dark side of employees’ bootlegging into account and explore the antecedents that will not bring destructive bootlegging.

Finally, the data source of this study is single, all of which are self-rated by employees. We have made efforts in this regard, that is, testing the common method variance following Podsakoff et al. (2003)’s suggestion and confirming that the common method variance did not significantly interfere with our conclusion. However, because of the limitations of our method, the influence of common method variance still cannot be eliminated. Hence, experimentation methods can be used further to test the robustness of the conclusions in the future.

## Data availability statement

The raw data supporting the conclusions of this article will be made available by the authors, without undue reservation.

## Author contributions

JJ contributed the central idea and collected the data. ZL contributed to writing the draft of the manuscript and analyzing the data. WL contributed to revising this manuscript and assisting in data analysis. JH contributed to revising this manuscript. All authors contributed to refining the article and approved the submitted version.

## Funding

This research was supported by the National Science Foundation of China, Project Nos. 72172032 and 71972032.

## Conflict of interest

The authors declare that the research was conducted in the absence of any commercial or financial relationships that could be construed as a potential conflict of interest.

## Publisher’s note

All claims expressed in this article are solely those of the authors and do not necessarily represent those of their affiliated organizations, or those of the publisher, the editors and the reviewers. Any product that may be evaluated in this article, or claim that may be made by its manufacturer, is not guaranteed or endorsed by the publisher.
